# Optimization and Analysis of Plates with a Variable Stiffness Distribution in Terms of Dynamic Properties

**DOI:** 10.3390/ma18092150

**Published:** 2025-05-07

**Authors:** Łukasz Domagalski, Izabela Kowalczyk

**Affiliations:** Department of Structural Mechanics, Lodz University of Technology, Politechniki 6, 93-590 Lodz, Poland; izabela.kowalczyk@dokt.p.lodz.pl

**Keywords:** genetic algorithms, optimization, band gaps, plates, eigenvalue problem, finite element method, dynamic analysis

## Abstract

This study investigates the optimization of thickness distribution in simply supported and cantilever plates to maximize gaps between adjacent natural frequencies. The research employs a genetic algorithm (GA) as the primary optimization tool, with the finite element method (FEM) integrated for structural dynamics analysis. The optimization process focuses on tailoring the plate thickness (stiffness) while maintaining fixed overall dimensions. The study considers square and rectangular plates with two boundary conditions: simply supported and cantilever. The optimization targets gaps between the first three natural frequencies. The GA-based optimizer demonstrates effectiveness in increasing the relative separation between neighboring natural frequencies, as defined by the fitness function. Compared to the reference individuals, the optimized individuals achieve objective function values from 0.25 to 2.5 times higher. The GA optimization tool is also compared with an alternative optimization tool achieving up to 35% better results. This research contributes to the field of structural dynamics by demonstrating the potential of genetic algorithms in optimizing plate designs for enhanced vibrational characteristics. Such optimization is particularly relevant in civil engineering, where plate elements are widely used, and where controlling dynamic properties can improve serviceability and reduce the risk of resonance under operational or environmental loads. The findings have implications for various engineering applications where controlling dynamic properties of plate structures is crucial.

## 1. Introduction

This study focuses on thickness distribution optimization of simply supported (SSSS) and cantilever plates (CFFF) to maximize the separation between specific neighboring natural frequencies. Thickness is used as a parameter directly influencing the mass and stiffness change. The approach used in this study, known as sizing optimization [1], involves modifying the dimensions of structural components to achieve superior performance. The analysis assumes the plates are made of a homogeneous material and follows the Kirchhoff–Love plate theory to model their behavior.

The phenomenon of band gaps in structural elements has been extensively investigated due to its relevance in numerous engineering applications. By adjusting band-gap properties, it becomes possible to influence the mechanical and vibrational characteristics of a structure, enabling improvements in its functionality and overall performance.

Several studies have explored different methodologies and frameworks for optimizing variable thickness plates. For instance, Deepika and Onkar developed a multicriteria design framework using the adaptive weighted sum method, integrating finite element methods and analytical sensitivity techniques to optimize the thickness distribution of isotropic plates under static and dynamic constraints [2]. Similarly, Xie et al. investigated the natural frequency and optimization design of nonlinear variable-thickness rectangular thin plates, demonstrating that a bidirectional stepped variable-thickness design could significantly enhance natural frequencies [3]. Banh et al. contributed to the field by proposing a topological optimization approach for Mindlin–Reissner plates with nonlinear variable thickness, emphasizing the effective utilization of materials to achieve optimal multi-material topologies [4]. Additionally, recent studies such as [5] used a two-field topology optimization algorithm for optimal design of variable-thickness fiber-reinforced plates. Ko and Solayev [6] and also proposed the topology optimization method applied to variable-thickness plates. Research using this method for plates with variable stiffness was also presented in [7] for a Reissner–Mindlin plate.

So far, many researchers have addressed the issue of plates with variable stiffness or thickness. The relationship between structural optimization and media with microstructure was explored by Kohn and Vogelius, who highlighted the potential of plates with rapid thickness variation to outperform traditional designs in terms of strength per unit volume [8]. Moruzzi et al. utilized adaptive finite elements for the free vibration analysis of plates with arbitrary thickness variations, demonstrating the efficiency of these elements in reducing computational demands while maintaining accuracy [9]. Lastly, Zhou and Liu focused on vibration control using composite variable-thickness wave absorbing damping plates, showing significant reductions in vibration amplitude without compromising structural integrity [10]. Additionally, Banh and Lee [11] presented a multi-material topology optimization approach for thin plates with variable thickness based on Kirchhoff plate theory using an alternating active-phase algorithm in conjunction with the block Gauss–Seidel method.

Numerical methods for solving band-gap problems in plate structures are diverse, each offering unique advantages in terms of accuracy and computational efficiency. The finite element method (FEM) is widely used, as demonstrated in numerous studies. For example, Ma et al. conducted the research on periodic piezoelectric micro-composite laminated plates, where it effectively models the band gap properties and supports feedback control systems for vibration management [12]. Similarly, FEM combined with Bloch’s theorem is employed to analyze 3D star-shaped resonant plate structures, providing detailed insights into band gap properties and enabling topological optimization for lower frequency band gaps [13]. The hybrid finite element-boundary element method (FE-BEM) is another approach, used to predict vibro-acoustic characteristics in periodic rib stiffened plates, offering a comprehensive analysis of bending wave propagation and noise reduction [14]. The asymptotic homogenization technique is yet another approach used to study transversal vibrations in thin periodic elastic plates. This method, combined with Bloch–Floquet analysis, helps predict band gaps by analyzing the dynamic surface density of the plate, particularly when it becomes negative, indicating band gap formation [15]. Additionally, the wave finite-element method adapted for polar coordinates is employed to study radially periodic plates, demonstrating its effectiveness in predicting bandgap characteristics and modeling complex structures [16].

In terms of optimization tools, metaheuristic algorithms, such as genetic algorithms (GA), ant colony optimization (ACO), and particle swarm optimization (PSO), have been recognized for their effectiveness in handling nonlinear design optimization problems with complex constraints and discrete design variables [17,18,19]. Study by Pyrz [20] applied genetic algorithms to optimize variable thickness plates under bending loads, achieving material distribution that minimizes the structural strain energy under constant volume. Garambois et al. employed a multi-objective genetic algorithm combined with a dynamic mixed plate finite element model to optimize plate thickness parameters under dynamic loads, achieving efficient solutions through the Kirchoff–Love plate theory, which allows for rapid adjustments in structural parameters [21]. Zhou focused on optimizing the lateral plates of a vibration screen using an improved genetic algorithm, incorporating sensitivity analysis to enhance optimization efficiency and reduce dynamic stress and weight, while also addressing noise reduction through modal experiments [22]. Furthermore, other methods such as artificial neural networks (ANN) combined with balancing composite motion optimization (BCMO) were utilized to optimize material distribution in functionally graded nanocomposite plates [23]. Comprehensive reviews by Raju [24] emphasize the growing role of quantum annealing and machine learning integrations in enhancing computational efficiency for large-scale structural optimizations. Neural network approaches, such as those developed by Chiba et al. [25], enable precise material composition tuning in functionally graded plates through deep learning-driven stress minimization.

Overall, the optimization and analysis of plates with variable stiffness distribution involve a diverse array of techniques and applications, each contributing to the enhancement of dynamic properties and structural performance. The integration of advanced numerical methods, such as finite element analysis and topology optimization, along with innovative design strategies, underscores the potential for significant advancements in this field, particularly in the field of engineering where dynamic performance is critical.

This study aims to determine the optimal stiffness distribution of linearly elastic plates to enhance their dynamic properties. The optimization focuses on the thickness as the design variable, while the plate’s overall dimensions remain fixed. Dynamic characteristics, particularly natural frequencies, are analyzed using the finite element method to ensure accurate modeling of vibrational behavior. To achieve optimal stiffness distribution, a genetic algorithm-based optimizer is employed, targeting the maximization of the relative separation between neighboring natural frequencies.

The present study introduces several key innovations and methodological improvements compared to the existing literature on the optimization of plate structures with variable stiffness or thickness. Conventional approaches mostly analyze periodic plate configurations through single-unit-cell models under idealized boundary conditions [16,17,21,22,26], they often neglect the realistic boundary conditions and finite dimensions of practical plate structures, limiting their applicability to real-world engineering scenarios. Presented research includes plates with actual boundary conditions (SSSS, CFFF) and finite plate dimensions, enabling the optimization of thickness distributions that account for edge constraints and real vibrational modes.

Furthermore, the GA implementation introduces problem-specific adaptations rarely detailed in prior works. Unlike studies employing genetic optimization tools with limited methodological transparency, our algorithm enforces symmetry constraints in chromosome construction based on the plate’s support type. This mirrors the symmetry/antisymmetry of vibration modes in symmetrically supported plates, ensuring physically consistent solutions and reducing the search space compared to unrestricted designs. Presented GA is specifically adapted to the problem of optimizing plate thickness distributions. Its mutation strategies and crossover mechanisms are explicitly defined, contrasting with implementations common in prior studies, where authors often provide only general descriptions of their optimization tools or do not elaborate on the specific implementation of genetic operators. This makes presented optimization process fully transparent and reproducible by other researchers.

The structure of this paper is as follows: Section 1 introduces the research topic and its context. Section 2 outlines the methods, including the theoretical background, the overall problem solution framework, the numerical solution approach, the optimization technique, and the application parameters used in the study. Section 3 presents the results, beginning with frequency optimization, followed by a detailed description of the optimization process and outcomes. This section also includes an analysis of the dynamic response, an examination of the natural forms (mode shapes), and a comparison with a gradient-based optimization tool. Section 4 discusses the findings and practical implications. Section 5 summarizes the main conclusions of the study. The references used throughout the paper are listed at the end.

## 2. Methods

### 2.1. Theoretical Background

**Figure 1:**
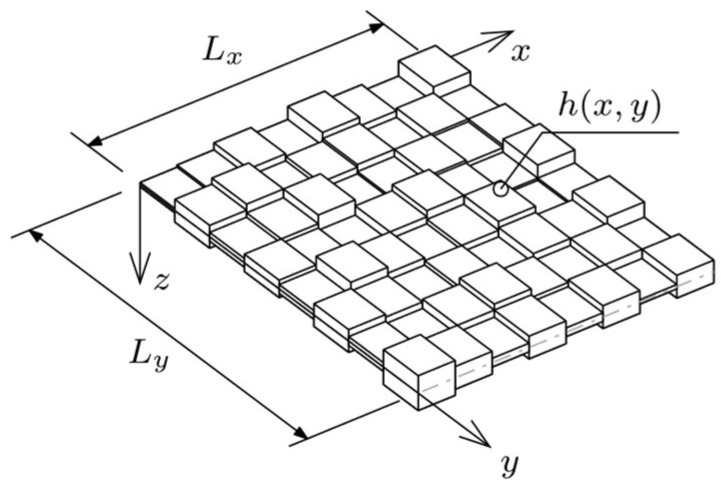
A computational model of the plate.

Let *Oxyz* be an orthogonal Cartesian coordinate system in the physical space. Considered a structural element in this system is a plate of dimensions *L_x_* × *L_y_*, which are parallel, respectively, to the *Ox* and *Oy* axes. The *Oz* is perpendicular to the mid-surface Π of the plate. The plate is of variable thickness *h* = *h*(*x*, *y*), and it is made of an isotropic linear-elastic material (Figure 1). To simplify the notation, it was assumed that: **x** = (*x*, *y*). Material properties are described by the following constants: Young’s modulus *E*, Poisson’s ratio *ν*, and density *ρ*. The plate is represented by a well-known model according to Kirchoff–Love theory for thin plates, which extends Euler–Bernoulli beam assumptions to thin plates with three fundamental kinematic constraints:

- Straight lines normal to the mid-surface remain straight and normal after deformation.
- The plate thickness remains constant during deformation.
- Transverse shear deformations are negligible, *ε_zz_* = 0.

The variational formulation of fundamental equations for this problem is briefly presented below, according to [27,28,29,30].

The analyzed element (plate) is considered in a certain finite time interval *T*, which is bound by *t*_0_ and *t*_1_, representing the initial and current moments, respectively, so the action functional is given by the following:

(1) $$A=\int_{t_{0}}^{t_{1}}Ldt,$$

The Langragian in Equation (1) is of the following form:

(2) L=K−W−qw+pw,

where w=w(x,t) stands for transverse deflection. In a general case, q=q(x,t) is a transverse load on the surface of the plate. The dissipative force *p* is assumed as follows, where c=c(x) stands for the damping coefficient:

(3) $$p=p(x,t)=c(x)\dot{w}(x,t).$$

The equations of motion can be obtained from the extended principle of stationary action (see [31]), formulated as follows:

(4) $$\delta A=\delta \left(\int_{t_{0}}^{t_{1}}Ldt\right)=\int_{t_{0}}^{t_{1}}\delta Ldt=0.$$

The strain or potential energy of deformed plate is equal to the following:

(5) $$W=\frac{1}{2}\underset{\Omega }{∭}\left(\sigma_{xx}\varepsilon_{xx}+\sigma_{yy}\varepsilon_{yy}+2\sigma_{xy}\varepsilon_{xy}\right)d\Omega ,$$

where $\Omega \equiv \left\{(x,z):x\in \prod ,-h(x)/2\leq z\leq h(x)/2\right\}$ denotes a bound region representing the undeformed plate. Strains *ε* are described by kinematic relations (6) and stresses *σ* by the stress–strain relations (7), according to Kirchoff–Love theory they are defined as follows:

(6) $$\begin{array}{l} w=w\left(x,t\right),\;u\left(x,z,t\right)=-z\frac{\partial w\left(x,t\right)}{\partial x},\;v=-z\frac{\partial w\left(x,t\right)}{\partial y}\\ \varepsilon_{xx}=\frac{\partial u}{\partial x}=-z\frac{\partial^{2}w}{\partial x^{2}},\;\varepsilon_{yy}=\frac{\partial v}{\partial y}=-z\frac{\partial^{2}w}{\partial y^{2}},\;\varepsilon_{xy}=\frac{\partial u}{\partial y}+\frac{\partial v}{\partial x}=-z\frac{\partial^{2}w}{\partial x\partial y},\\ \varepsilon_{zz}=\frac{\partial w}{\partial z}=0,\;\varepsilon_{xz}=\frac{\partial u}{\partial z}+\frac{\partial w}{\partial x}=0,\;\varepsilon_{yz}=\frac{\partial v}{\partial z}+\frac{\partial w}{\partial y}=0, \end{array}$$

(7) $$\sigma_{xx}=\frac{E}{1-\nu^{2}}\left(\varepsilon_{xx}+\nu \varepsilon_{yy}\right),\;\sigma_{yy}=\frac{E}{1-\nu^{2}}\left(\varepsilon_{yy}+\nu \varepsilon_{xx}\right),\;\sigma_{xy}=\frac{E}{1-\nu^{2}}\varepsilon_{xy},$$

where *ν* stands for Poisson’s ratio. Displacements variables *u* and *v* used in Equation (6) stand for the displacements of a point in the plate along the *x* and *y* axes, respectively. According to the Kirchhoff–Love theory, the transverse deflection w does not depend on the through-thickness coordinate *z*. In consequence transverse strain components *ε_xz_*, *ε_yz_* and *ε_zz_* are equal to zero.

Substituting the kinematic relations (6) and stress–strain constitutive laws (7) into the potential energy formulation (5) yields the final strain energy expression (8):

(8) $$W=\frac{E}{2\left(1-\nu^{2}\right)}\underset{\Omega }{∭}z^{2}\left\{{\left(\frac{\partial^{2}w}{\partial x^{2}}\right)}^{2}+{\left(\frac{\partial^{2}w}{\partial y^{2}}\right)}^{2}+2\nu \frac{\partial^{2}w}{\partial x^{2}}\frac{\partial^{2}w}{\partial y^{2}}+2\left(1-\nu \right){\left(\frac{\partial^{2}w}{\partial xy}\right)}^{2}\right\}d\Omega .$$

Considering only the transverse motion, the kinetic energy of the plate can be expressed as follows:

(9) $$K=\iint_{\prod }\frac{1}{2}\mu (x){\dot{w}}^{2}d\prod ,$$

where the overdot above *w* stands for the derivative taken with respect to time *t* and μ(x) is the mass density of the plate material per unit area:

(10) $$\mu =\mu (x)=\int_{-h(x)/2}^{h(x)/2}\rho dz.$$

Substitution of aforementioned Equations (1)–(10) and integrating by parts leads to Equation (11):

(11) $$\nabla^{2}(D\nabla^{2}w)-(1-\nu )\left(\frac{\partial^{2}D}{\partial y^{2}}\frac{\partial^{2}w}{\partial x^{2}}-2\nu \frac{\partial^{2}D}{\partial x\partial y}\frac{\partial^{2}w}{\partial x\partial y}+\frac{\partial^{2}D}{\partial x^{2}}\frac{\partial^{2}w}{\partial y^{2}}\right)+c\dot{w}+\mu \ddot{w}=q(x,t),$$

where D=D(x) is varying flexural stiffness, and $\nabla^{2}$ is the Laplace operator, given by Equation (12). The *f*(**x**) stands for a twice-differentiable real-valued function:

(12) $$\nabla^{2}f(x)=\frac{\partial^{2}f(x)}{\partial x^{2}}+\frac{\partial^{2}f(x)}{\partial y^{2}}.$$

Equation (11) constitutes the fourth-order governing equation of motion for the out-of-plane displacement of thin plate with variable thickness [30].

### 2.2. Problem Solution Framework

#### 2.2.1. Numerical Solution

The natural frequencies and mode shapes, representing the plate’s free vibration characteristics, as well as the amplitudes of forced vibrations, were determined by solving Equation (11) with the finite element method (FEM). The equations were formulated based on [32]. The plate was discretized using four-node rectangular elements.

The generalized equation of motion in FEM dynamics analysis is given by the following:

(13) $$M\ddot{q}(t)+C\dot{q}(t)+Kq(t)=F(t),$$

where **M**, **C**, **K** are matrices of mass, damping and stiffness, respectively. The remaining components of Equation (13) depend on time *t*, where **F**(*t*) is vector of external loads, and **q**(*t*) is displacement vector. First time derivative of displacement $\dot{q}(t)$ is velocity, and second derivative $\ddot{q}(t)$ stands for acceleration. The matrices of mass **M**, damping **C** and stiffness **K** for entire structure can be defined as follows:

(14) $$M=\sum_{e}M^{\left(e\right)},\;C=\sum_{e}C^{\left(e\right)},\;K=\sum_{e}K^{\left(e\right)},$$

where the superscript (*e*) denotes single finite element *e*. To define all components in a presented formula, let the stress–strain relation be expressed in a matrix form:

(15) $$\sigma^{\left(e\right)}=D^{\left(e\right)}\varepsilon^{\left(e\right)},$$

where **σ**^(*e*)^ and **ε**^(*e*)^ stand for stress and strain vectors, respectively, **D**^(*e*)^ is the stress–strain relation matrix.

Strain Formula (6) in a matrix form, can be expressed as follows:

(16) $$\varepsilon^{\left(e\right)}={\left[\begin{array}{c} \varepsilon_{xx}\\ \varepsilon_{yy}\\ \varepsilon_{xy} \end{array}\right]}^{\left(e\right)}=-z{\left[\begin{array}{c} \kappa_{xx}\\ \kappa_{yy}\\ \kappa_{xy} \end{array}\right]}^{\left(e\right)}=-z\kappa^{\left(e\right)},$$

where **κ**^(*e*)^ stands for plate’s curvature. Similarly, stress–strain relations (7) in a matrix form are defined as follows:

(17) $$\sigma^{\left(e\right)}={\left[\begin{array}{c} \sigma_{xx}\\ \sigma_{yy}\\ \sigma_{xy} \end{array}\right]}^{\left(e\right)}=-D^{\left(e\right)}\varepsilon^{\left(e\right)}=-zD^{\left(e\right)}\kappa^{\left(e\right)},$$

where

(18) $$D^{\left(e\right)}=\frac{E}{1-\nu^{2}}\left[\begin{array}{ccc} 1 & \nu & 0\\ \nu & 1 & 0\\ 0 & 0 & (1-\nu )/2 \end{array}\right].$$

Unit shear forces on the edge of the plate element, in the middle plane, replacing the linearly varying stresses at the height of the element are defined as follows:

(19) $$\begin{array}{l} m^{\left(e\right)}={\left[\begin{array}{c} m_{xx}\\ m_{yy}\\ m_{xy} \end{array}\right]}^{\left(e\right)}=\int_{-h/2}^{h/2}\sigma^{\left(e\right)}zdz=-\int_{-h/2}^{h/2}D^{\left(e\right)}\kappa^{\left(e\right)}z^{2}dz=-{\bar{D}}^{\left(e\right)}\kappa^{\left(e\right)}=-{\bar{D}}^{\left(e\right)}B^{\left(e\right)}v^{\left(e\right)}, \end{array}$$

where

(20) $${\bar{D}}^{\left(e\right)}=\frac{Eh^{3}}{12(1-\nu^{2})}\left[\begin{array}{ccc} 1 & \nu & 0\\ \nu & 1 & 0\\ 0 & 0 & (1-\nu )/2 \end{array}\right].$$

In this case, *h* is also thickness, but it is constant for individual plate element *e*. **B**^(*e*)^ stands for linear strain matrix of elasticity of element *e*, and **v**^(*e*)^ is matrix of geometrical parameters for individual element.

Based on the principle of stationary action (4) presented in Section 2.1, the stress–strain relations (15) and strain-displacement relations (16), the mass, damping, and stiffness matrices in the generalized equation of motion (13) can be expressed as follows:

(21) $$K^{\left(e\right)}=\int_{A^{\left(e\right)}}B^{(e)T}{\bar{D}}^{\left(e\right)}B^{\left(e\right)}dA^{\left(e\right)}.$$

**K**^(*e*)^ is stiffness matrix for element *e*. **V**^(*e*)^ is matrix of static parameters for individual element, and *A*^(*e*)^ stands for area of the element *e*.

The mass matrix **M**^(*e*)^ and damping matrix **C**^(*e*)^ for individual plate element *e* can be defined as follows:

(22) $$M^{\left(e\right)}=\int_{A^{\left(e\right)}}\rho^{\left(e\right)}N^{T(e)}N^{\left(e\right)}dA^{\left(e\right)},$$

(23) $$C^{\left(e\right)}=\int_{A^{\left(e\right)}}\eta^{\left(e\right)}N^{T(e)}N^{\left(e\right)}dA^{\left(e\right)},$$

where **N**^(*e*)^ is the shape functions vector of element *e*, **ρ**^(*e*)^ is mass density matrix, and *η*^(*e*)^ is damping parameter of element *e*. Here, the damping matrix was defined according to Rayleigh damping model, commonly used in numerous studies [33,34]:

(24) C=αM+βK,

where *α* and *β* are the Rayleigh coefficients.

In the case of forced harmonic vibrations, the external force can be expressed as follows:

(25) $$F(t)=F_{0}(t)\cos(\omega t).$$

Application of the multimodal approach for the forced vibration analysis leads to the following solution:

(26) $$q=a_{C}\cos\left(\omega t\right)+a_{S}\sin\left(\omega t\right),$$

where parameter *ω* is angular frequency, and coefficients **a**_C_ and **a**_S_ are defined as follows:

(27) $$\begin{array}{c} a_{C}={\left\{\left(K-\omega^{2}M\right)+\omega^{2}C\left[{\left(K-\omega^{2}M\right)}^{-1}C\right]\right\}}^{-1}F_{0},\\ a_{S}=\omega {\left(K-\omega^{2}M\right)}^{-1}C{\left\{\left(K-\omega^{2}M\right)+\omega^{2}C\left[{\left(K-\omega^{2}M\right)}^{-1}C\right]\right\}}^{-1}F_{0}. \end{array}$$

In this particular optimization problem, since we consider free vibrations, the damping matrix is equal to zero, and no external forces are involved. Thus, Equation (13) takes the following form:

(28) $$M\ddot{q}(t)+Kq(t)=0.$$

The solution can be expressed as follows:

(29) $$q=q_{a}\sin(\omega t),$$

where **q**_a_ stands for eigenvector. It can be expressed as follows, where *m* is number of degrees of freedom:

(30) $$q_{a}=\left[q_{a,1},q_{a,2},\ldots ,q_{a,m}\right].$$

Assuming that eigenvalue *λ* is equal to *ω*^2^, Equation (29) can be written as follows:

(31) $$\left(K-\lambda M\right)q_{a}=0.$$

Only the result where **q**_a_ ≠ 0 is considered, so to determine eigenvalues and eigenvectors, the following condition has to be met:

(32) $$\det\left|K-\lambda M\right|=0.$$

#### 2.2.2. Optimization Technique

In this work, genetic algorithm was used as the main optimization tool. GAs are optimization techniques inspired by Darwin’s theory of natural selection. Genetic operators and strategies are explained according to the following books and works: [35,36,37,38]. The algorithm presented in this work was developed specifically for its needs and adapted to the presented optimization problem. The algorithm was created using the Python 3.9.13 programming language supported mainly by following libraries: NumPy, SciPy and Matplotlib.

The plate was divided into *n*_el_ elements. Every individual element is described by numerical value-thickness *h_i_*, where *i* = 1, 2, …, *n*_el_. An element, or more precisely, its thickness value, constitutes a single gene. The thickness is variable parameter—different for individual elements and subject to changes during the optimization process. As the elements are connected to create a plate, the genes are grouped to create a chromosome. Therefore, in GA a single plate is represented by a chromosome. Going further, a group of chromosomes constitutes a population.

The first step in the GA procedure is to create the initial population. This population consists of *n*_pop_ individuals (chromosomes) and is generated randomly using the pseudo-random number generator build in Python’s library—in this case, NumPy’s ‘random’ function was used, to create the beta distribution with shape parameters *a*, *b* in the probability density function. Values of the generated genes (thicknesses *h*_i_) are limited by minimum and maximum thresholds. The algorithm always creates individuals that are symmetrical and, depending on their support type, cantilever plates are symmetrical to the axis perpendicular to the supported edge, and simply supported plates are symmetrical to both axes. This procedure reduces the computational costs of the whole process.

In the next step, the randomly generated initial population is evaluated in terms of the objective function. In this work, the objective function describes the relative difference between two adjacent natural frequencies *ω_k_* and *ω_k_*_+1_, where *k* stands for number of natural vibration frequency. The function describes the relative width of the bandgap. Adapting dimensionless values with a maximum value of one is advantageous and convenient in terms of numerical calculations. The objective function was defined as follows:

(33) $$\max\Delta \omega_{k}=\max\frac{\omega_{k+1}-\omega_{k}}{\omega_{k+1}}.$$

Values of natural frequencies are determined using FEM built-in to the algorithm, according to the solutions presented in the previous subsection. The algorithm calculates solutions of a given mechanical problem (in this case, natural frequencies of individual plates) and then substitutes them into (33) to perform evaluation. Individuals described by the highest values of objective function are then subjected to the genetic operators. The algorithm creates a vector where the evaluated individuals are arranged according to their rating.

To enhance population diversity and reduce the likelihood of premature convergence, mutations are employed in the algorithm, as they introduce controlled, random changes in the individuals’ genes. Specifically, the best individuals are modified using ‘non-uniform mutation’ for all genes in the chromosome. This allows for introducing random changes in the chosen gene, with the nature of these changes (narrowing or widening of the value) being controlled by the random variable. Conversely, individuals rated lower by the objective function are subjected to ‘roll mutation’ or ‘hard-swap mutation’. The first type involves shifting all genes in the chromosome by a random number of places, while the second one swaps a gene to its maximal or minimal permissible value.

The next step is initializing a new set of parents (one pair of parents = two chromosomes) according to the roulette wheel selection mechanism. The number of parents is pre-defined in the algorithm, and it is selected for crossover by sampling from the cumulative probabilities. In this selection mechanism there are specific restrictions to prevent form dawning chromosomes that are too similar in a single pair.

Offspring from selected pairs of parents (chromosomes) are created through crossover operations. Four types of crossovers were used in this study: ‘one-point crossover’ (OPCX), ‘two-point crossover’ (TPCX), ‘random-point crossover’ (RPCX), and ‘blend crossover’ (BLXa). The probability of each of them being applied in the algorithm is equal to 25%. The OPCX is a process that involves selecting a random crossover point in the parents’ chromosomes and exchanging parts of their genes to create the offspring. TPCX is similar type of genetic operation, but instead one point of division, there are two random crossover points in the parents’ chromosome. In RPCX a few random genes are crossed between two parents to generate offspring. The last one, BLXa also blends parent A and parent B. It randomly generates new individuals by blending the genes within a specified range based on a defined blending factor.

Finally, the offspring resulting from these operations are evaluated and sorted according to the criteria imposed by the objective function. Excess individuals are removed to maintain the appropriate population size. The population is updated with new individuals, and the genetic algorithm procedure is repeated.

After completing the whole optimization process using GAs, the results were compared with results obtained using another optimization tool–the ‘minimize’ function from the SciPy library in Python. This function offers a variety of optimization algorithms, and in this study, the Sequential Least Squares Programming (SLSQP) method was utilized due to its effectiveness in handling constrained nonlinear optimization problems, which aligns well with the problem of optimizing plate thickness for maximizing frequency gaps.

#### 2.2.3. Application Parameters

We consider a plate of *E* = 205 GPa, Poisson’s ratio *ν* = 0.3, and density *ρ* = 7850 kg/m^3^. The dimensions of the plate are *L_x_* × *L_y_* = 1 m × 1 m or *L_x_* × *L_y_* = 2 m × 1 m. For both sizes, two types of plate boundary conditions were considered: simply supported on all edges (SSSS) and cantilever (CFFF) plate. Gaps between adjacent vibration frequencies were investigated in the range of *k* from 1 to 3. That amounts to 12 considered cases in following study. The plate was divided into elements for FEM analysis, but also for GA optimization.

**Figure 2:**
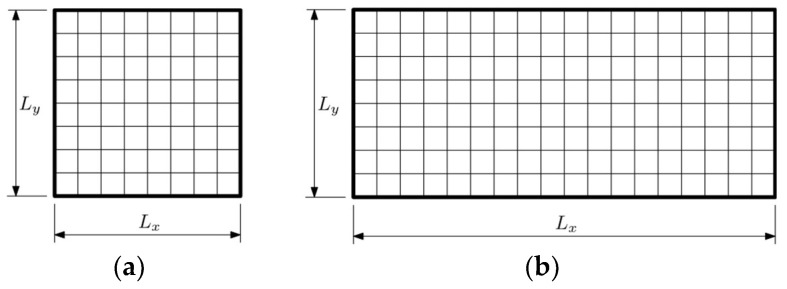
Considered plates divided into finite elements: (**a**) *L_x_* × *L_y_* = 1 m × 1 m with 64 elements, and (**b**) *L_x_* × *L_y_* = 1 m × 1 m with 128 elements.

The described GA was applied to optimize the thickness distribution of plates divided into *n*_el_ = 64 elements, square plates, and *n*_el_ = 128, rectangular plates (Figure 2). Each element’s thickness was constrained between *h*_min_ = 2.5 × 10^−3^ m and *h*_max_ = 10 × 10^−3^ m. The beta distribution used to initialize the population was defined with shape parameters *a* and *b*—both equaled to 0.5. Initial population size was set to *n*_pop_ = 360, and the number of populations varied depending on the case, typically between 30 and 40. The population size and selection parameters were tailored to ensure computational efficiency and algorithm convergence.

**Figure 3:**
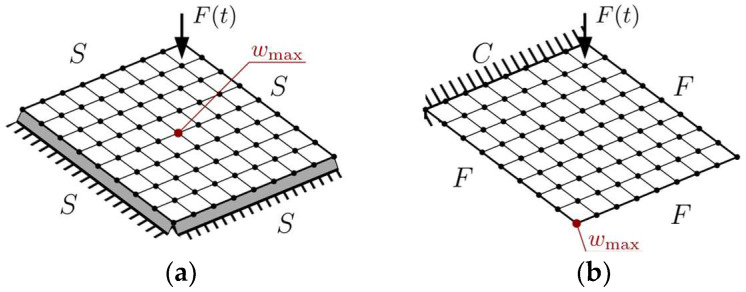
Forced vibration analysis: (**a**) SSSS plate, and (**b**) CFFF plate.

Moreover, forced vibration analysis was performed for optimized plates. The maximum displacement *w*_max_ was analyzed for both the simply supported and cantilever plates. The excitation force *F*(*t*) was located in the node closest to the support, but depending on the boundary conditions, the displacement was investigated for different nodes. They are presented in Figure 3.

In dynamic analysis, the Rayleigh coefficients *α* and *β* in damping model are equal to 2∙10^−6^ and 3∙10^−5^ respectively.

The first step in following research was to optimize the randomly generated plate in terms of issues connected to natural vibrations using FEM and Gas. Second step was to analyze the obtained results (optimized plates) in terms of two problems. First, an analysis of natural vibrations and a comparison to check if the objective function was fulfilled were performed. Second, an analysis of forced vibrations using FEM and comparison of reference individuals with optimized individuals were performed.

## 3. Results

### 3.1. Frequency Optimization

#### 3.1.1. Optimization Process

The optimization process is a key stage of this study, as it directly determines how the genetic algorithm iteratively refines the thickness distribution of the plate to maximize the targeted frequency gaps. In this subsection, we present a detailed visualization and analysis of the population evolution during selected optimization runs, providing insight into the convergence behavior and diversity of solutions generated by the GA at various stages of the process.

**Figure 4:**
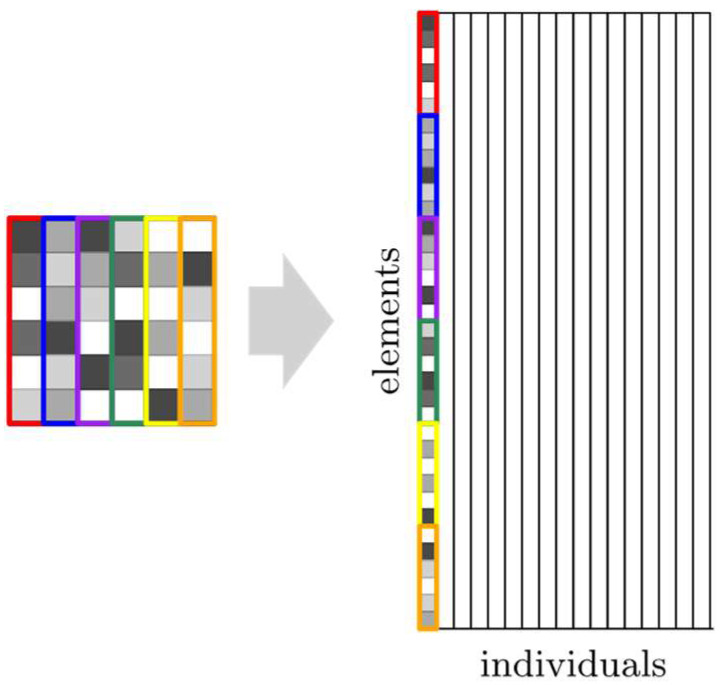
Explanation of the results presented.

**Figure 5:**
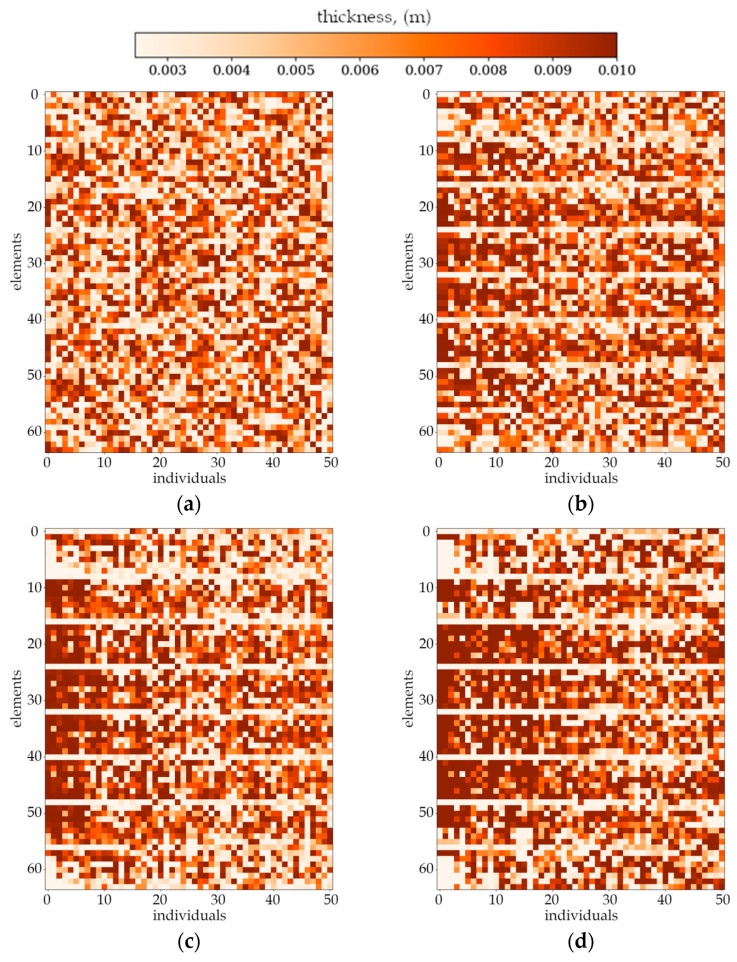
Evolution of the example population. From left to right: population number (**a**) 0, (**b**) 4, (**c**) 10 and (**d**) 30. Optimization in terms of Δ*ω*_1_ for CFFF, *L_x_* × *L_y_* = 1 × 1.

**Figure 6:**
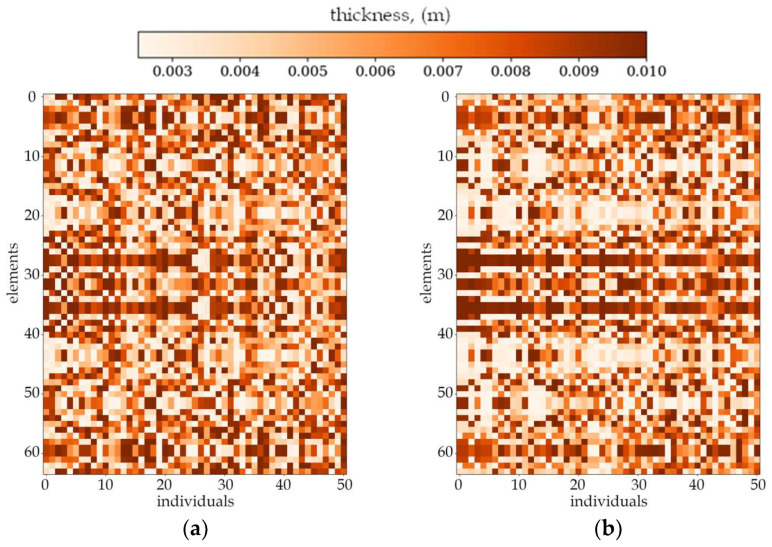

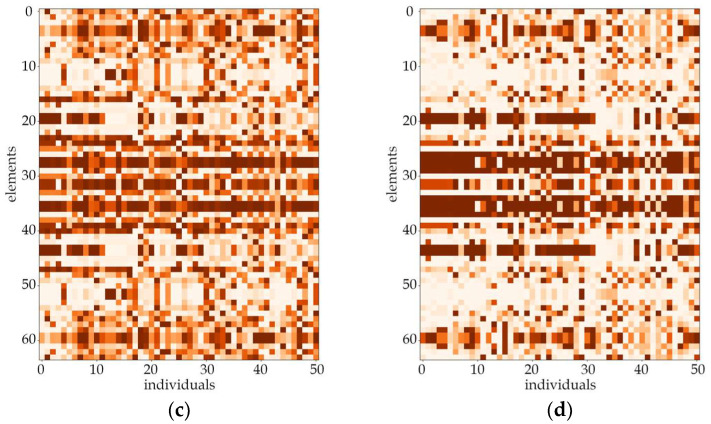
Evolution of the example population. From left to right: population number (**a**) 0, (**b**) 4, (**c**) 10 and (**d**) 40. Optimization in terms of Δ*ω*_3_ for SSSS, *L_x_* × *L_y_* = 1 × 1.

As shown in Figure 4, each column of the plot represents one individual in the population (one column on the plot is one plate divided into elements). The representation of the whole population by single plot gives a clearer comparison of changes between successive populations and graphically illustrates the efficiency of the optimization algorithm. It also presents every individual in the selected population to illustrate how individuals within a single population differ from one another at the various stages of an evolution process. Figure 5 and Figure 6 show the selected cases of optimization. For each case, there are four chosen populations presented as individual plots.

The randomness in the initial population generated randomly by beta distribution is visible in each case. First noticeable changes in the mass distribution for individual plates appear around 4th to 6th population. At this point, it becomes evident that neighboring individuals achieve a similar thickness distribution (darker and lighter bands begin to form on the whole population plot). Changes in the mass distribution for individuals occur more gradually as the population numbers increase.

In most cases, the evolution process ends by the 40th or 50th population. This is due to a constraint in the algorithm that breaks calculations if the objective function value of the best individual differs by less than 1% from the best individual in the previous population.

**Figure 7:**
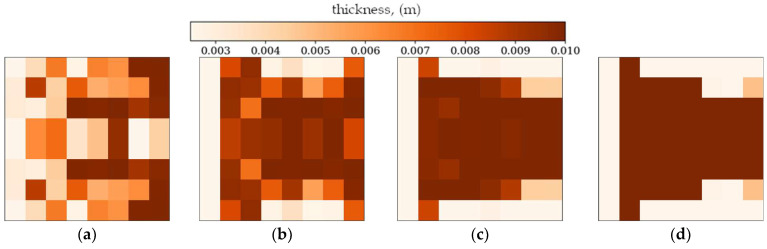
The best individuals from chosen populations. From left to right: population number (**a**) 0, (**b**) 4, (**c**) 10 and (**d**) 30. Optimization in terms of Δ*ω*_1_ for CFFF, *L_x_* × *L_y_* = 1 × 1.

**Figure 8:**
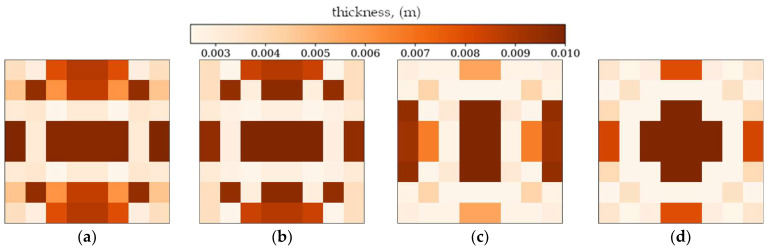
The best individuals from chosen populations. From left to right: population number (**a**) 0, (**b**) 4, (**c**) 10 and (**d**) 40. Optimization in terms of Δ*ω*_3_ for SSSS, *L_x_* × *L_y_* = 1 × 1.

**Figure 9:**
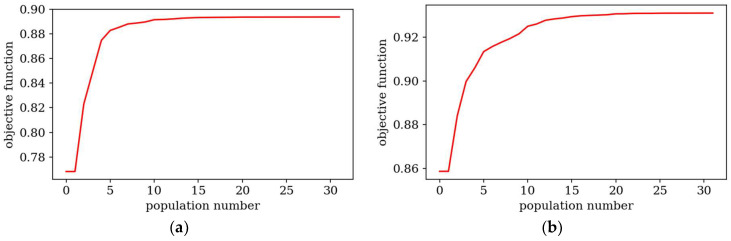
Objective functions for the best individual in subsequent populations. Optimization in terms of Δ*ω*_1_ for CFFF: (**a**) *L_x_* × *L_y_* = 1 × 1, (**b**) *L_x_* × *L_y_* = 2 × 1.

**Figure 10:**
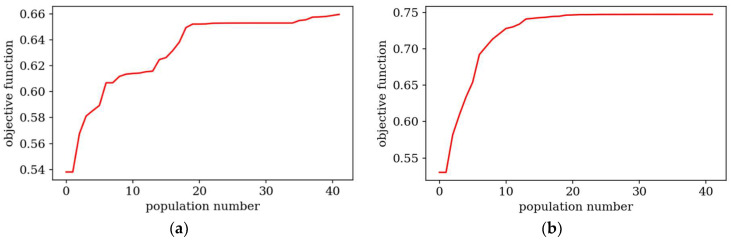
Objective functions for the best individual in subsequent populations. Optimization in terms of Δ*ω*_3_ for SSSS: (**a**) *L_x_* × *L_y_* = 1 × 1, (**b**) *L_x_* × *L_y_* = 2 × 1.

Figure 7 and Figure 8 present the best individuals from chosen populations, for cases corresponding to those shown in Figure 5 and Figure 6. The optimization algorithm uses symmetry of the horizontal axis (for CFFF) and symmetry of the horizontal and vertical axes (for SSSS) to shorten the computation time. Figure 9 and Figure 10 show the fitness function values obtained by the best individual in each population. Figure 9 and Figure 10 correspond to Figure 5 and Figure 6, respectively.

#### 3.1.2. Optimization Outcomes

**Figure 11:**
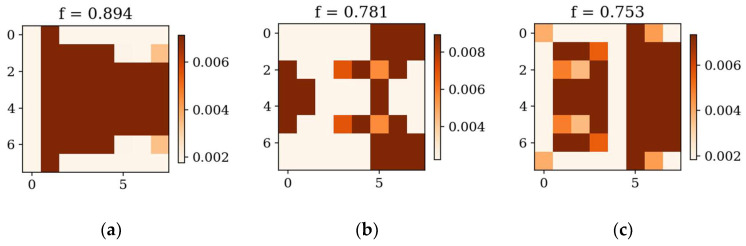
The best individuals for CFFF, *L_x_* × *L_y_* = 1 × 1. Optimization in terms of (**a**) Δ*ω*_1_, (**b**) Δ*ω*_2_ and (**c**) Δ*ω*_3_.

**Figure 12:**
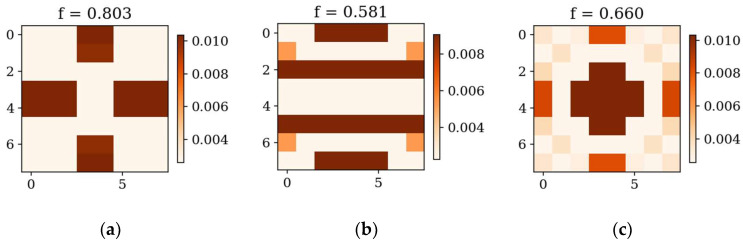
The best individuals for SSSS, *L_x_* × *L_y_* = 1 × 1. Optimization in terms of (**a**) Δ*ω*_1_, (**b**) Δ*ω*_2_ and (**c**) Δ*ω*_3_.

**Figure 13:**
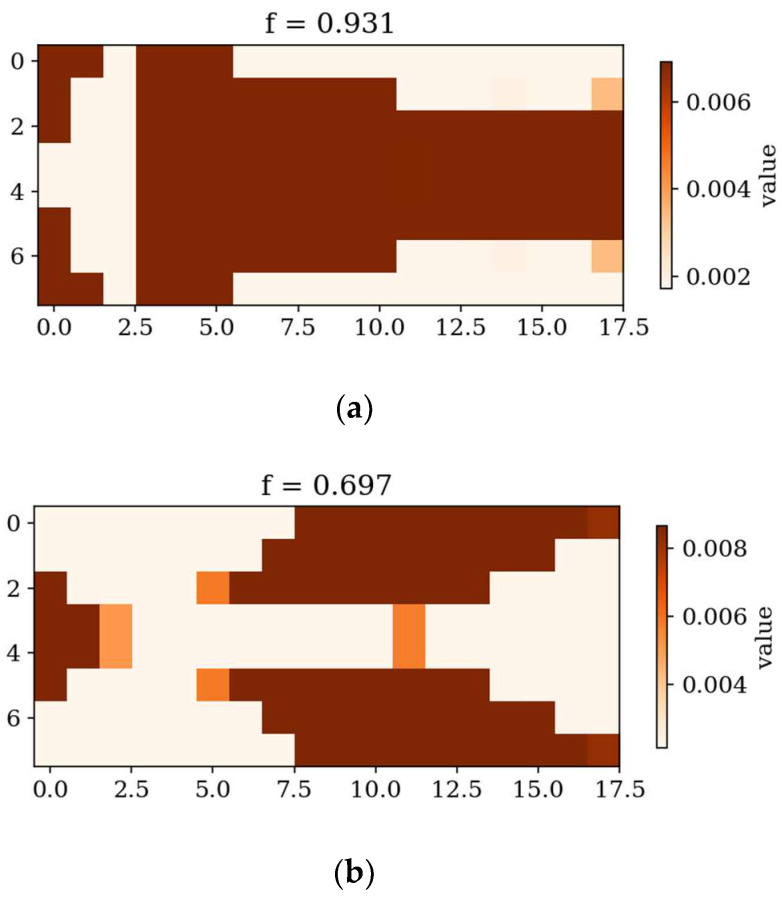

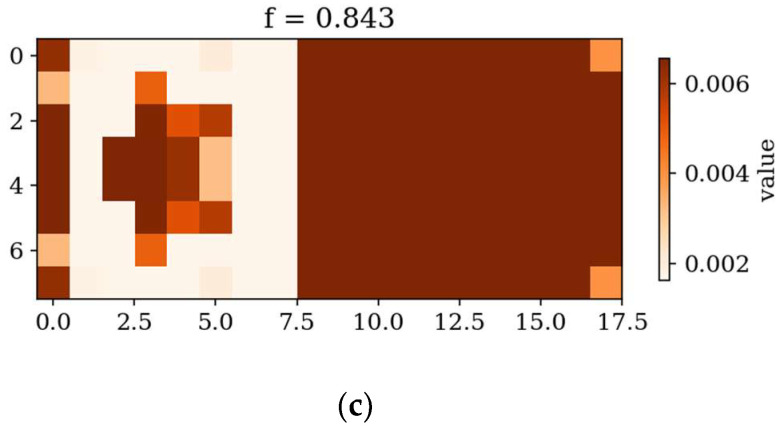
The best individuals for CFFF, *L_x_* × *L_y_* = 2 × 1. Optimization in terms of (**a**) Δ*ω*_1_, (**b**) Δ*ω*_2_ and (**c**) Δ*ω*_3_.

**Figure 14:**
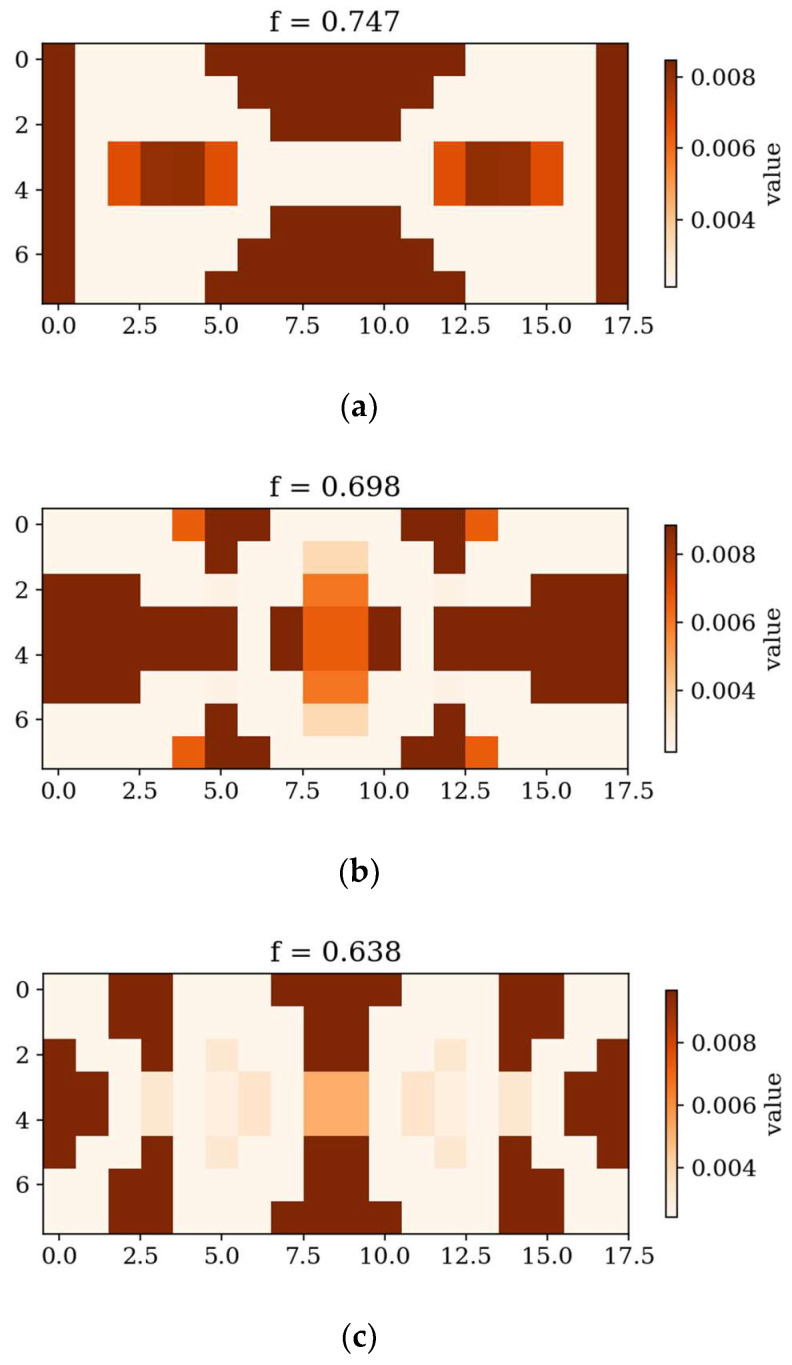
The best individuals for SSSS, *L_x_* × *L_y_* = 2 × 1. Optimization in terms of (**a**) Δ*ω*_1_, (**b**) Δ*ω*_2_ and (**c**) Δ*ω*_3_.

In the following subsection, the best individuals for each case of optimization are presented and described. These results highlight the thickness distributions achieved through the optimization process for plates with different boundary conditions CFFF and SSSS, see Figure 11, Figure 12, Figure 13 and Figure 14. Darker areas indicate regions with a greater thickness concentration.

The fitness function values achieved by the best individuals are also presented in Table 1 and Table 2, which include the percentage differences in the fitness function values for optimized and non-optimized (reference) individuals. A reference individual is a homogeneous plate whose thickness equals the average thickness of the corresponding optimized individual (the best-performing plate from the final population).

### 3.2. Dynamic Response Analysis

**Figure 15:**
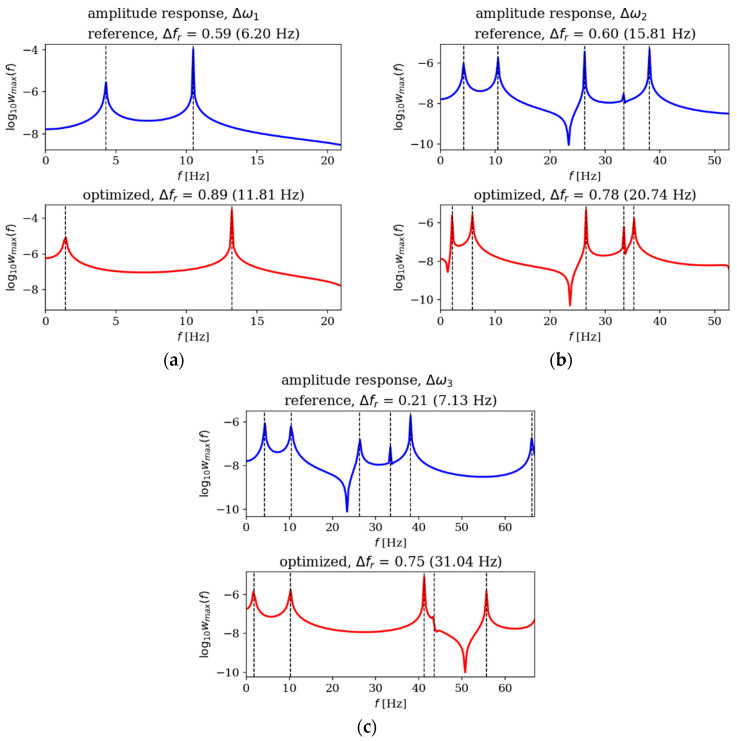
Amplitude response comparison: reference vs. optimized plates for CFFF, *L_x_* × *L_y_* = 1 × 1. Optimization in terms of (**a**) Δ*ω*_1_, (**b**) Δ*ω*_2_ and (**c**) Δ*ω*_3_.

**Figure 16:**
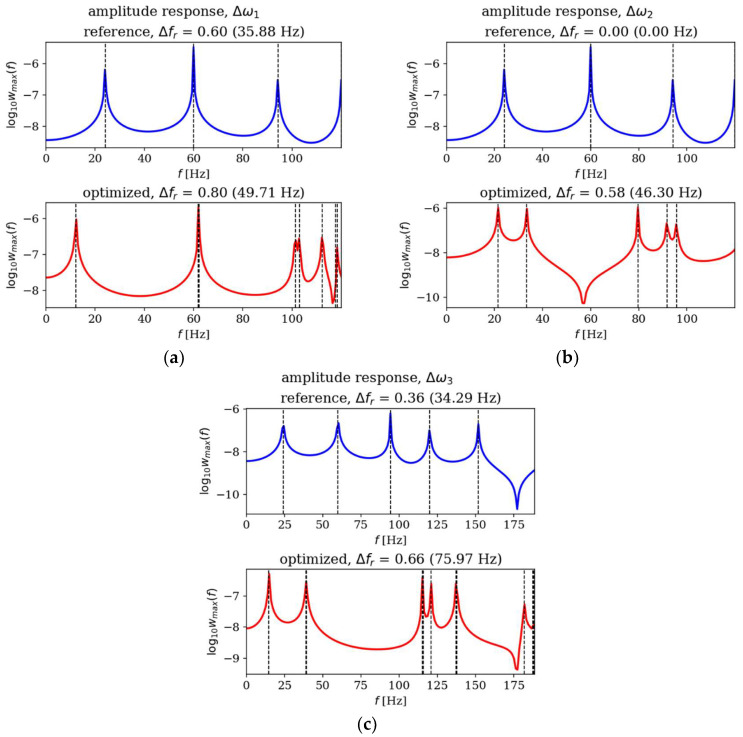
Amplitude response comparison: reference vs. optimized plates for SSSS, *L_x_* × *L_y_* = 1 × 1. Optimization in terms of (**a**) Δ*ω*_1_, (**b**) Δ*ω*_2_ and (**c**) Δ*ω*_3_.

**Figure 17:**
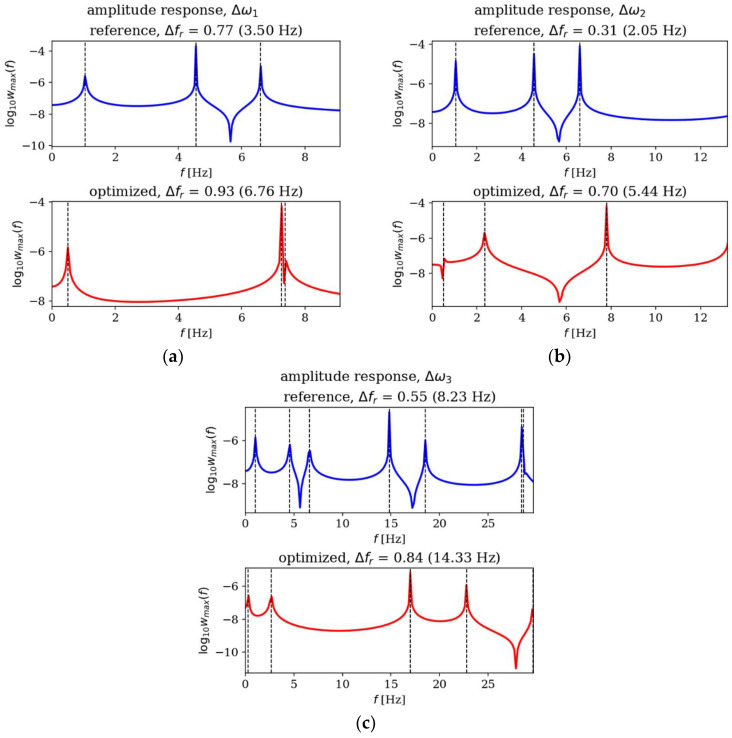
Amplitude response comparison: reference vs. optimized plates for CFFF, *L_x_* × *L_y_* = 2 × 1. Optimization in terms of (**a**) Δ*ω*_1_, (**b**) Δ*ω*_2_ and (**c**) Δ*ω*_3_.

**Figure 18:**
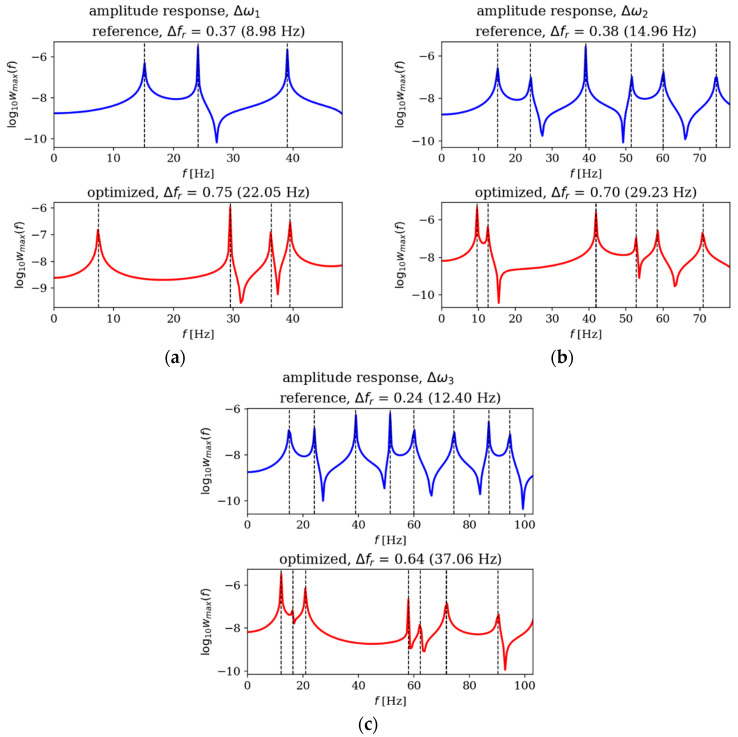
Amplitude response comparison: reference vs. optimized plates for SSSS, *L_x_* × *L_y_* = 2 × 1. Optimization in terms of (**a**) Δ*ω*_1_, (**b**) Δ*ω*_2_ and (**c**) Δ*ω*_3_.

In this subsection, the amplitude responses of plates before and after optimization are analyzed and compared. Figure 15, Figure 16, Figure 17 and Figure 18 present the results for all cases. The response plots illustrate the relationship between vibration amplitude and frequency values, with reference individuals included in all cases to isolate the effects of thickness distribution optimization.

### 3.3. Natural Forms

**Figure 19:**
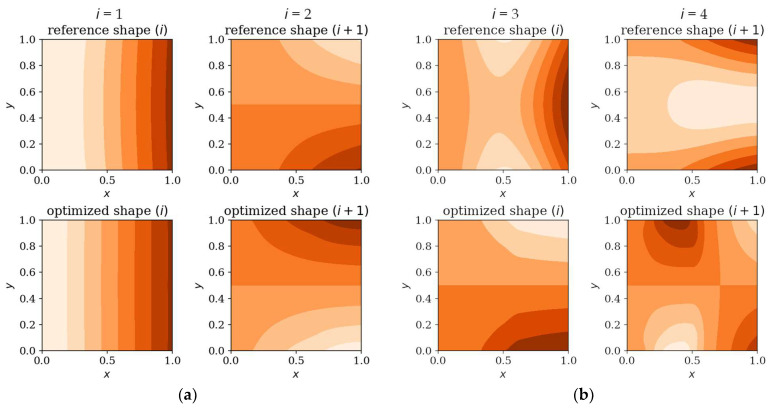
Natural forms comparison: reference vs. optimized CFFF plates, *L_x_* × *L_y_* = 1 × 1. Optimization in terms of (**a**) Δ*ω*_1_, (**b**) Δ*ω*_3_.

**Figure 20:**
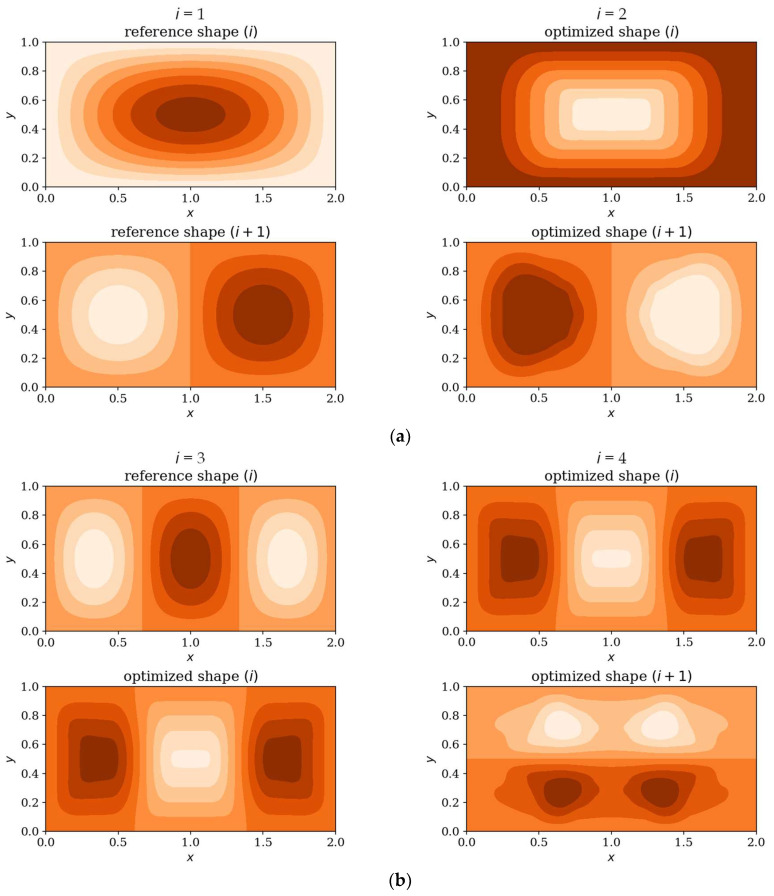
Deflection shape comparison: reference vs. optimized SSSS plates, *L_x_* × *L_y_* = 2 × 1. Optimization in terms of (**a**) Δ*ω*_1_, (**b**) Δ*ω*_3_.

In the following subsection, the natural mode shapes for CFFF and SSSS plates before and after optimization are presented (chosen cases presented in Figure 19 and Figure 20). The results highlight how the optimization process influences the distribution and symmetry of mode shape patterns, providing deeper insight into the structural response of the plates under various boundary conditions.

### 3.4. Gradient-Based Optimization Tool

**Figure 21:**
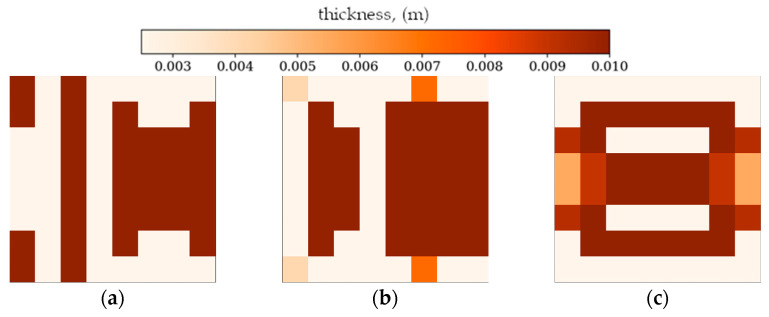
Optimized square plates obtained using SciPy: (**a**) Δ*ω*_1_ for CFFF, (**b**) Δ*ω*_3_ for CFFF, and (**c**) Δ*ω*_2_ for SSSS.

**Figure 22:**
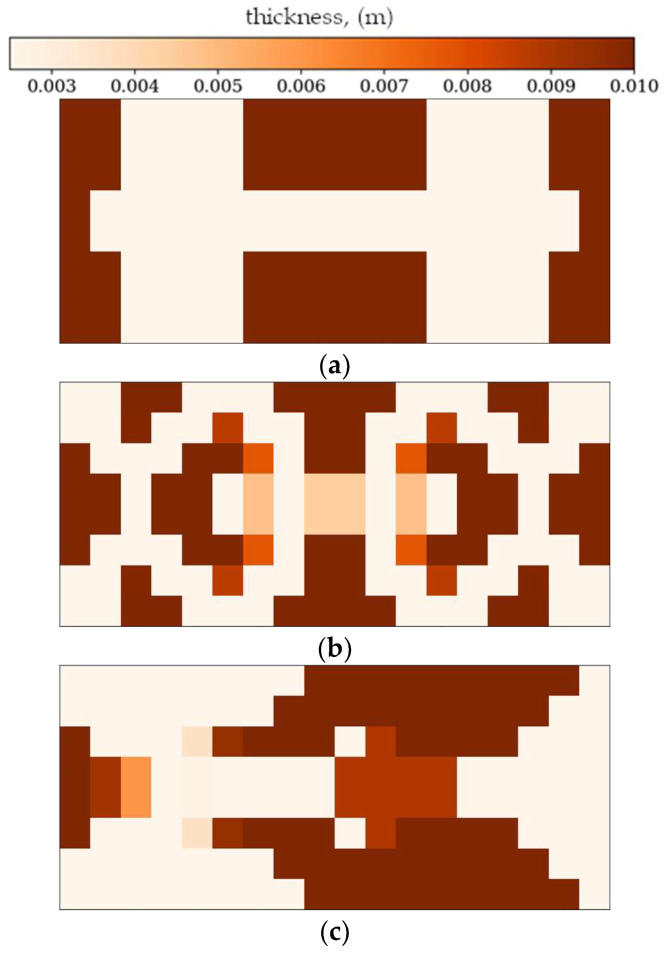
Optimized rectangular plates obtained using SciPy: (**a**) Δ*ω*_1_ for SSSS, (**b**) Δ*ω*_3_ for SSSS, and (**c**) Δ*ω*_2_ for CFFF.

In the following subsection, optimization results obtained from alternative, gradient-based optimization tool–SciPy minimize–are presented. The SciPy optimization demonstrated a reduced computational time compared to the GA. However, a comparison of the optimized objective function values reveals some trade-offs. Figure 21 and Figure 22 present the optimized thickness distributions obtained using SciPy for various plate configurations and optimization targets.

The results are also presented in numerical form in Table 3 and Table 4. They contain percentage comparisons of the objective function values for square (Table 3) and rectangular plates (Table 4).

## 4. Discussion

This study demonstrates that genetic algorithm-based plate thickness distribution optimization allows maximizing the spacing between adjacent natural frequencies and thus influences plates dynamic response.

Based on the progress of the optimization process graphically illustrated by Figure 5, Figure 6, Figure 7, Figure 8, Figure 9 and Figure 10, the optimization is most dynamic during its initial phases. Naturally, this is reflected in the objective function values achieved by individual plates in successive populations. It can also be observed that during optimization in terms of lower natural frequencies, the evolution process usually ends up faster than in the case of optimization for higher frequencies.

Optimization process outcomes visualized as patterns (Figure 11, Figure 12, Figure 13 and Figure 14) correspond to the natural vibration modes targeted during optimization, with darker regions indicating areas of higher thickness concentration. By analyzing these distributions, it is possible to observe how the algorithm adapted the structural layout to fulfill the fitness function. The patterns formed on the plates correspond to the natural vibration modes. These areas play a critical role in shaping the vibration frequencies and modes, reducing amplitudes at nodal lines or stabilizing parts of the plate that are not intended to move significantly. For lower frequencies (e.g., Δ*ω*_1_), the patterns are simpler and cover larger regions, mainly within the extreme thicknesses of the plate. For higher frequencies (Δ*ω*_2_ and Δ*ω*_3_), the patterns become more complex, reflecting a greater number of nodal lines in the vibration modes. Moreover, the plates surfaces have areas with more variable thicknesses than after the Δ*ω*_1_ optimization.

The rectangular plates exhibit elongated patterns of thickness distribution, aligning with their aspect ratio. In contrast to the square plates, the optimization process concentrates stiffness along the longer dimension in regions critical to stabilizing vibrations, reflecting the influence of the plate’s geometry on the distribution. For the CFFF plate, *L_x_* × *L_y_* = 2 × 1, higher thickness concentrations are observed near the supported edge. The results confirm that the optimization algorithm effectively tailors the stiffness distribution to match the unique dynamic behavior of plates with different geometries. The elongated patterns observed in rectangular plates align well with the expected deformation shapes of the longer structure during vibration.

From analyzing the numerical results in Table 1 and Table 2, it can be seen that optimization significantly increased the fitness function values for all frequencies (Δ*ω*_1_, Δ*ω*_2_ and Δ*ω*_3_) for both cantilever and simply supported plates, indicating that higher frequencies are probably more sensitive to optimization. For both square and rectangular plates with both types of boundary conditions, the largest increase was observed in the optimization in terms of Δ*ω*_2_ and Δ*ω*_3_. In the case of square cantilever-supported plates (CFFF), the increase in fitness function was more substantial than for simply supported plates (SSSS). This suggests that optimization is more effective in asymmetrically supported conditions, where there is greater potential for improvement. These observations highlight that both plate geometry and support conditions influence the efficiency of the optimization process.

The optimization process undoubtedly influenced the dynamic response of the analyzed plates, regardless of their boundary conditions and dimensions. In the case of the square SSSS plate (Figure 16), the optimized individuals exhibited higher amplitude values compared to the reference individuals within the frequency ranges targeted by the optimization. For rectangular SSSS plates (Figure 18), a more pronounced reduction in amplitude is noticeable within the frequency range for which the optimization was carried out. Conversely, for the rectangular CFFF plate, an increase in amplitudes is observed in the case of optimized individuals (Figure 17).

Figure 19 and Figure 20 shows that the optimization process affects the natural modes, making them more complex and diverse, which is more visible when optimizing in terms of higher frequencies; at low frequencies the changes are almost imperceptible. The optimized individuals exhibited thickness patterns aligned with vibration mode symmetries such as elongated configurations in rectangular plates and edge-thickened profiles in cantilever (CFFF) plates.

Finally, a comparison of the GA and SciPy optimization results revealed that the performance of both methods depends on the complexity of the optimization target and boundary conditions. For simpler cases, such as optimization in terms of lower frequencies, the differences in objective function values between GA and SciPy were minimal (e.g., 1.12% for the CFFF square plate). However, for more complex cases (optimization in terms of Δ*ω*_2_ and Δ*ω*_3_) or for plates with simply supported boundary conditions (SSSS), GA consistently outperformed SciPy, achieving significantly higher objective function values (e.g., 34.85% improvement for the SSSS square plate, in terms of Δ*ω*_3_).

The results (Table 3 and Table 4) indicate that while SciPy offers a rapid means of achieving a reasonable solution, the GA can, in some instances, achieve superior results, although at a higher computational cost. The SciPy’s SLSQP method, being a gradient-based technique, tends to converge to local optima, especially in complex solution spaces, while the GA’s metaheuristic-based approach allows for a more thorough exploration of the solution space, increasing the likelihood of finding the global optimum.

## 5. Conclusions

In the presented work, the application of genetic algorithms for the thickness distribution optimization of simply supported and cantilever plates was investigated, with a focus on maximizing the gaps between adjacent natural frequencies. The results obtained were analyzed to formulate the following conclusions:

- The application of GAs proved to be effective in optimizing the plates for maximizing the gaps between natural frequencies. Optimized structural elements exhibited increased gaps between adjacent natural frequencies, according to the defined fitness function.
- Unlike prior studies focusing on infinite periodic plates or single-unit-cell models, this work introduces a GA tailored for finite plates with realistic boundary conditions. By enforcing symmetry constraints in chromosome encoding and integrating FEM-based dynamic analysis, the algorithm achieves up to 34.85% higher objective function values than gradient-based method (SLSQP) for complex cases.
- The GA’s mutation strategies and crossover mechanisms are explicitly defined, addressing a gap in the prior literature. This transparency enables reproducibility and adaptation for similar structural optimization problems.
- Faster convergence in the optimization process is observed when optimizing for lower natural frequencies compared to higher frequencies.
- For lower frequencies (Δ*ω*_1_), the thickness distribution is simpler, covering larger regions, whereas for higher frequencies (Δ*ω*_2_, Δ*ω*_3_), the patterns are more complex with varied thicknesses.
- The optimized plates show a structure related to vibration modes, which is especially visible at higher frequencies. This correlation could enable targeted stiffening of nodal regions.
- The optimization process had a complex impact also on the amplitude response.

Future research will focus on using finite elements with variable stiffness and imposing constraints on the dimensional differences between adjacent elements, allowing for further optimization of stiffness distribution. Experimental results will also be obtained to verify the dynamic characteristics of the optimized elements. Additionally, the research will include an analysis of genetic algorithm methods, examining their parameters and potentially developing custom variants of the optimization procedures.

To confirm the reliability and practical applicability of the optimized thickness distributions obtained in this study, experimental validation is essential and could also be a part of future research related to this topic. The most direct approach involves fabricating physical plate specimens with the optimized variable-thickness profiles, as well as corresponding reference (homogeneous) plates with matching average thickness. Plates could be manufactured using 3D printing or layered assembly, ensuring geometric accuracy and material consistency. The measurements should include an excitation of examined elements with the use of specialized exciters and then the registration of the dynamic response of composite structures measured with accelerometers.

## Figures and Tables

**Table 1:** Comparison of fitness function values between the optimized and non-optimized individuals—square plate.

*L_x_* × *L_y_* = 1 × 1					
*K*	The Individual	Plate CFFF		Plate SSSS	
		Δ*ω_k_*	Difference	Δ*ω_k_*	Difference
1	reference	0.59	50.85%	0.60	33.33%
	optimized	0.89		0.80	
2	reference	0.60	30.00%	0.00	-
	optimized	0.78		0.58	
3	reference	0.21	257.14%	0.36	83.33%
	optimized	0.75		0.66	

**Table 2:** Comparison of fitness function values between the optimized and non-optimized individuals—rectangular plate.

*L_x_* × *L_y_* = 2 × 1					
*K*	The Individual	Plate CFFF		Plate SSSS	
		Δ*ω_k_*	Difference	Δ*ω_k_*	Difference
1	reference	0.77	20.78%	0.37	102.70%
	optimized	0.93		0.75	
2	reference	0.31	125.81%	0.38	84.21%
	optimized	0.70		0.70	
3	reference	0.55	52.73%	0.24	166.67%
	optimized	0.84		0.64	

**Table 3:** Comparison of the objective function values obtained by the GA and SciPy optimization procedures—square plate.

*L_x_* × *L_y_* = 1 × 1						
*k*	Plate CFFF			Plate SSSS		
	Δ*ω_k_*		Difference	Δ*ω_k_*		Difference
	GA	SciPy		GA	SciPy	
1	0.89	0.88	1.12%	0.80	0.78	2.50%
2	0.78	0.75	3.85%	0.58	0.38	34.48%
3	0.75	0.77	2.67%	0.66	0.43	34.85%

**Table 4:** Comparison of the objective function values obtained by the GA and SciPy optimization procedures—rectangular plate.

*L_x_* × *L_y_* = 2 × 1						
*k*	Plate CFFF			Plate SSSS		
	Δ*ω_k_*		Difference	Δ*ω_k_*		Difference
	GA	SciPy		GA	SciPy	
1	0.93	0.89	4.30%	0.75	0.74	1.33%
2	0.70	0.69	1.43%	0.70	0.70	≪1.00%
3	0.84	0.77	8.33%	0.64	0.62	3.13%

## Data Availability

The original contributions presented in this study are included in the article. Further inquiries can be directed to the corresponding author.
